# Intra-Rater and Inter-Rater Reliability of Pressure Pain Algometry of the Sural and Tibial Nerves in Asymptomatic Elite Youth Footballers

**DOI:** 10.3390/sports9090132

**Published:** 2021-09-18

**Authors:** Daniel Richards, Simon Jones, Josh Jeffery, Matthew Lowe, Mark Godwin, Matthew Willett

**Affiliations:** 1 School of Sport, Exercise and Rehabilitation Sciences, University of Birmingham, Birmingham B15 2TT, UK; richard1@hope.ac.uk; 2 School of Sport and Exercise Science, Liverpool Hope University, Liverpool L16 9JD, UK; 3 Everton Football Club, Finch Farm, Liverpool L4 4EL, UK; s.jones27@nhs.net (S.J.); josh.r.jeffery@gmail.com (J.J.); matty.lowe@hotmail.co.uk (M.L.); 4 School of Health, Sport, and Food, University College Birmingham, Birmingham B3 1QH, UK; M.Godwin@ucb.ac.uk; 5 Centre of Precision Rehabilitation for Spinal Pain, University of Birmingham, Birmingham B15 2TT, UK

**Keywords:** reliability, pain pressure threshold, football, Tibial nerve, Sural nerve

## Abstract

Ankle injuries are highly prevalent in elite youth footballers and increase the mechanosensitivity of the local neural tissue, which may predispose athletes to re-injury and prolong rehabilitation periods. Increased neural mechanosensitivity presents clinically as altered pain pressure thresholds (PPTs) which are measured with pressure algometry. The purpose of this study was to determine the intra-rater and inter-rater reliability of PPTs of the ankle neural tissue in asymptomatic elite youth football players. Three raters utilised a digital algometer to evaluate the PPTs of the Sural and Tibial nervous tissue at the ankle of elite youth male footballers. Intraclass correlation coefficients (ICCs) with 95% confidence intervals (CI) were calculated to assess intra-rater and inter-rater reliability and Bland–Altman figures were plotted to enable visual evaluation of measurement error with a significance level of *p* < 0.05. Thirty-four players (16–18 years old) were assessed. Excellent intra-rater (Tibial ICC 0.88 (0.76–0.94); Sural ICC 0.89 (0.79–0.95)) and good inter-rater reliability (Tibial ICC 0.66 (0.40–0.82); Sural 0.71 (0.50–0.85)) was demonstrated. Bland–Altman plots demonstrated low levels of measurement error. Pressure algometry can be utilised clinically to accurately evaluate the PPTs of the Tibial and Sural nervous tissue at the ankle in asymptomatic elite male youth footballers.

## 1. Introduction

Football is the world’s most popular sport [1] with over 12,000 elite youth players playing in the professional leagues in England [2]. Musculoskeletal injuries are highly prevalent in elite youth football [3], where the incidence is reported to be between 2.4 [4] and 4.8 [5] injuries per 1000 training hours, and may result in physical pain, reduced function, and psychological distress for players. Ineffective assessment and management may prolong rehabilitation of musculoskeletal injuries and reduce game play and contribute to a potential loss of earnings for the player and revenue for club [6]. In elite youth football, prolonged musculoskeletal injuries are particular problematic, as they reduce opportunities for career development and may impact on future professional contracts [7].

The ankle is the most common region of musculoskeletal injury (16–20% of all injuries) and reinjury (>25% of total injuries) in elite youth football [5]. Hyper-inversion and hyper-eversion ankle injuries damage the lateral and medial ligament complexes, respectively [8], and increase the mechanosensitivity of the local neural tissues (e.g., Sural and Tibial nerves) [9]. Importantly, increased mechanosensivity of ankle neural tissue may predispose players to develop chronic ankle instability [10] and increase the likelihood of injury recurrence and prolonged rehabilitation periods [11]. Therefore, the assessment of the mechanosensitivity of neural structures local to the ankle should be included in a routine clinical assessment of elite youth footballing players by the club medical staff.

Assessment of neural mechanosensitivity is primarily determined by evaluating pain pressure thresholds (PPTs) through algometry [9]. PPTs are defined as the minimum amount of mechanical stimulus applied to a tissue for it to be perceived as painful [11]. To date, two studies [11,12] have evaluated the reliability of PPTs of the lower-limb peripheral nervous tissue using pain pressure algometry. Walsh and Hall (2009) [12] calculated excellent (mean Intraclass Correlation Coefficient (ICC) range: Tibial 0.87–0.96; Sciatic: 0.92–0.96; Common Peroneal 0.85–0.91) inter-rater reliability of PPTs in patients with low back and related leg pain. Fingleton et al., (2014) [11] calculated good (mean ICC: Femoral 0.69; Tibial 0.84) to excellent (mean ICC: Common Peroneal 0.84; Sciatic 0.90) intra-rater reliability and fair (mean ICC: Tibial 0.56), good (mean ICC: Common Peroneal: 0.70), and excellent (mean ICC: Sciatic 0.75; Femoral 0.82) inter-rater reliability respectively in an asymptomatic population. However, both studies assessed the hip (Sciatic and Femoral) [11] or knee (Tibial and Common Peroneal) [11,12] with no study to date having evaluated the mechanosensitivity of the ankle’s nervous tissue. Therefore, the aim of this study was to determine the intra-rater and inter-rater reliability of PPTs, as assessed with a pain algometer, of the Sural and Tibial nerves in asymptomatic elite youth football players.

## 2. Materials and Methods

### 2.1. Study Design

This study was granted ethical approval by the University of Birmingham (CM260318-1) and is reported in line with the Guidelines for Reporting Reliability and Agreement Studies (GRRAS) document [13]. The medical staff and elite youth male footballers at the testing club were covered by ‘Sempris’ and ‘Healthpartners’ insurance providers, respectively.

### 2.2. Participants

Prior to recruitment a power calculation was conducted. Using the guidance outlined by Walter et al., (1998), the minimum sample size required to achieve significance (*p* < 0.05) was 33 participants [14]. A convenience sample of male elite youth players (U16/17/18) at a Premier League football club in the North West of England were invited to take part in the study (see Table 1 for participant eligibility criteria). To avoid coercion, the invitation was delivered verbally and in written format by a non-testing researcher. The email address and phone number of the primary researcher (DR) were listed on all written documents. Potential participants were given two weeks to decide whether they would participate and no incentive to take part. Prior to study DR verbally explained the testing procedure again and answered any queries. All participants read the participant information sheet and signed the informed consent form prior to participating in the study. Where players were under the age of 18 at the time of recruitment, their parents or legal guardians provided this authorisation.

### 2.3. Procedure

#### 2.3.1. Familiarisation and Researchers

Three raters were involved in pain pressure testing. At the time of testing, Rater 1 (DR) was a chartered Physiotherapist with 7 years’ experience working in elite sport and had previous experience using pressure pain algometry in clinical practice and for research purposes; Rater 2 (SJ) was a qualified medical doctor with 6 years’ post graduate experience and 2 years working in elite sport; Rater 3 (JJ) was a Sports Therapist with 4 years’ post graduate experience working in elite sport. The fourth researcher (ML), was a qualified Sports Therapist with 1 year of post graduate experience, who acted as a non-testing researcher. Prior to data testing, DR conducted a one-hour training session with an algometer for the other raters which included identification of neural points to be examined, the full testing protocol and recording of date. A further practice test was undertaken on asymptomatic participants not involved in the study immediately prior to data collection [15]. The studies initial aim was to determine the PPT values of the Tibial, Sural, and Superficial Peroneal nervous tissue at the ankle. However, during the familiarisation session, the raters struggled intermittently to locate the Superficial Peroneal nervous tissue as it emerged from the lateral compartment of the lower-leg anterior to the Lateral Malleolus [16]. This is consistent with other studies which suggest the Superficial Peroneal Nerve can only be accurately located in just over 50% of ankles [17]. Therefore, the decision was made to exclude the Superficial Peroneal nerve from the study, leaving the Tibial and Sural nerves for assessment.

#### 2.3.2. Randomisation, Location, and Timing

All participants were assigned a participant number based on their order of recruitment. A random sequence generator (random.org) (accessed on 30 April 2019) was used to allocate participants to a rater and determine which neural structure would be assessed first (i.e., Tibial or Sural). Testing was conducted in the medical room of the Premier League football Club. The total time participants were involved in testing was approximately 30 min on two occasions, two weeks apart. To minimise the effect of fatigue, each testing session was done at the same time, which was prior to training, and 3 days following a competitive match.

#### 2.3.3. Recording Instrument and Application

The Wagner digital algometer (Wagner, FPX 10, Greenwich, CT, USA) was used to assess the pain pressure thresholds of the Tibial and Sural nervous tissue at the ankle [16]. This device has demonstrated excellent (mean ICC: 0.91) inter-rater reliability when utilised by newly trained raters in contemporary studies [18]. The algometer had a probe size of 1 cm^2^ and pressure was applied manually at a rate of 30 kPa/s until the participant’s PPT was reached [11]. Thirty kPa/s was chosen to give participants enough time to respond to the stimulus and avoid additional pressure after the PPT had been reached [11,19].

#### 2.3.4. Testing

**Figure 1:**
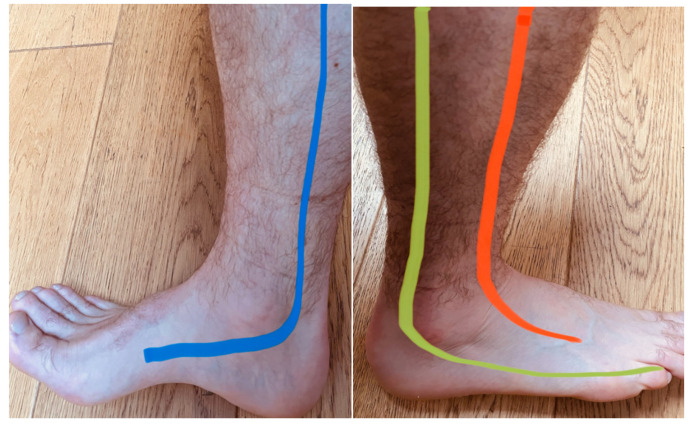
Anatomy of the peripheral distribution of the Tibial (Blue), Superficial Peroneal (Orange) and the Sural (Yellow) nervous tissue.

**Figure 2:**
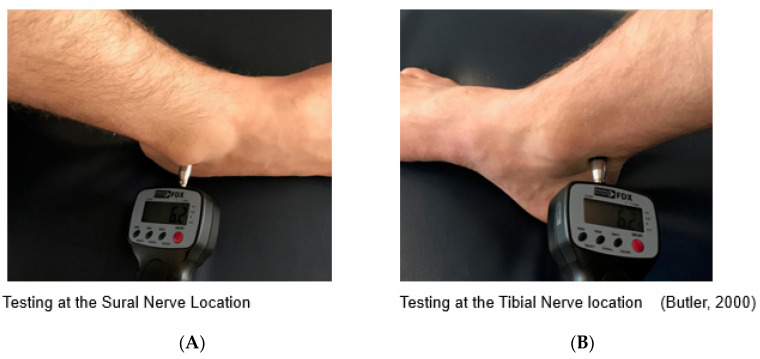
Pain Pressure Threshold testing locations of the Sural (**A**) and Tibial nerves (**B**).

Prior to data collection, a point on the wrist was assessed with the algometer to familiarise participants with the testing procedure [12]. Participants were instructed to lay supine on a medical plinth and their exposed non-dominant knee bent to 90 degrees and their foot plantar side down on the medical plinth [16]. The non-dominant leg was chosen for testing to avoid any potential irritation to participants dominant ankle prior to training. The Tibial and Sural nerves were located 2 cm posterior to the medial malleolus and 1 cm inferior to the lateral malleolus and marked with a felt pen by the non-testing researcher [16] The anatomy of the ankle nervous tissue and testing point location can be seen in Figure 1 and Figure 2, respectively.

Three PPT measurements were taken and the mean calculated [11,18]. Algometer pressure was applied perpendicular to the skin [15] with a 30 s rest between consecutive measures to avoid sensitising local tissue and temporal summation [20]. Standardised verbal instructions of “I am about to apply pressure to your skin. You must tell me the moment the sensation alters from non-unpleasant pressure to slightly unpleasant pain” were used by assessors prior to testing [18]. The statement was printed on a poster above each medical plinth for rater reference. The term “slightly unpleasant” was chosen to conform to the International Association for the Study of Pain (IASP) definition of pain threshold [21] to avoid testing participants pain tolerance [15]. Once the participants’ pain pressure threshold was reached, the pressure was released.

#### 2.3.5. First Testing Session: Inter-Rater Reliability

For pragmatic reasons, three participants were tested at three testing stations in one room simultaneously. Participants remained on the same medical plinth and the rater rotated between them. To reduce rater-bias and ensure participant blinding, the digital screen was faced downwards during testing then lifting towards the non-testing researcher after the PPT had been reached. The recorder inputted PPT values directly to an excel spread sheet and did not offer any verbal feedback. All medical plinths were over 2 m apart to enhance rater blinding.

#### 2.3.6. Second Testing Session: Intra-Rater Reliability

Rater 1 (DR) tested each participant a second time two weeks after the initial testing session. The same non-testing researcher from the first testing session was present and marked up participants and recorded PPT values in an identical manner as previously outlined.

### 2.4. Statistical Analysis

Statistical analysis was conducted using IBM SPSS version 24 for Macintosh (SPSS Inc., Armonk, NY, USA) software with all data presented as mean ± standard deviation (SD). The mean and standard deviation of PPT measurements were calculated for the Sural and Tibial nervous tissue. The Standard Error of Measurement (SEM) was calculated to evaluate the variability between scores which could be due to measurement error following the equation SEM = S√1 − ICC, where ‘S’ refers to the pooled standard deviation (SD). To make reasonable judgements whether a real change has occurred, the minimal detectable change (MDC) was calculated using the formula: (1.96)√2(SEM^2^) [22].

Intra-rater (within participant; between testing sessions 1 and 2) and inter-rater reliability (within participant; between rater in session 1) were calculated using two-way analysis of variance (random effects model) [23]. Intraclass correlation coefficients (ICCs) with 95% confidence intervals (CI) were calculated to assess intra-rater and inter-rater reliability. ICC values were interpreted as; excellent: >0.75; good: 0.6–0.75; fair: 0.4–0.59; poor: <0.4 [24]. Bland–Altman figures were plotted to enable visual evaluation of agreement consistency for intra-rater and inter-rater reliability testing of the Tibial and Sural nervous tissue. Proportional bias was assessed with linear regression and a significance level of *p* < 0.05 was utilised for all analyses.

## 3. Results

### 3.1. Participants

Thirty-nine players were invited and all agreed to participants in the study. Five were excluded due to current or previous musculoskeletal injuries, meaning thirty-four elite youth football players aged between 16 and 18 years (mean 17.0 years ± 1.0 years) participated in the study (Table 2).

### 3.2. Mean and Range of PPT Values

Mean PPT values ranged from 34.00–47.47 kPa at the Tibial and 31.59–50.23 kPa in the Sural nervous tissue respectively across three raters. PPT values including mean, standard deviation and minimum and maximum values (displayed n kPa) are detailed in Table 3.

### 3.3. Reliability

Excellent intra-rater reliability was found for the Tibial (0.88 [95% CI 0.76, 0.94]) and Sural (0.89 [0.79, 0.95]) nervous tissue respectively. Good inter-rater reliability was calculated across the three raters for the Tibial (0.66 [0.40, 0.80]) and Sural (0.71 [0.50, 0.80]) nerves respectively. Intra-rater and Inter-rater ICCs, SEM, and MDC values are shown in Table 4. Visual evaluation of the plots demonstrates low levels of measurement error with no obvious increases at different pressure thresholds. Linear regression suggested all Bland–Altman plots were free of proportional bias except the intra-rater testing of the Sural nervous tissue (*p* < 0.01). The Bland–Altman plots for intra-rater and inter-rater reliability and linear regression results are presented in Figure S1.

## 4. Discussion

### 4.1. Intra Class Correlation Coefficients

This is the first study to investigate the intra-rater and inter-rater reliability of PPT assessment of the Tibial and Sural nervous tissue with a widely used pressure algometer in elite youth footballers. The results demonstrated excellent intra-rater reliability (Sural ICC: 0.88 [95% CI 0.76–0.94]; Tibial ICC: 0.88 [0.79–0.95]) and good inter-rater reliability (Sural ICC: 0.72 [0.50–0.85]; Tibial ICC: 0.66 [0.40–0.82]) for both anatomical locations.

The lower ICC values found in inter-rater compared with intra-rater reliability is consistent with other studies that have examined PPTs of the nervous tissue in other body segments [11,15]. These results may be explained by natural variation in rater application of algometry testing due to variations in point location, angle of application, and the force exerted by each researcher with the algometer against each participants’ skin. Our inter-rater ICC values and 95% confidence interval ranges were comparable to Fingleton et al., (2014) (0.56–0.90) [11]. However, Walsh and Hall (2009) [12] (0.85–0.96) and Sterling et al., (2000) (0.92–0.97) demonstrated higher ICC values when testing nervous tissue at the hip/knee [12] and median/radial/ulnar nerve trunks [13] respectively. While the aforementioned studies followed a standardised PPT assessment protocol and outlined rater experience, only Fingleton et al., (2014) described rater training prior to testing. However, this was not reported with sufficient detail to enable replication and a definitive link between rater training and reliability of PPT testing with pressure algometry cannot be established. Furthermore, authors disagree on the training needs of raters prior to pressure algometry testing to establish adequate reliability [15,25].

In their study with pre-registration physiotherapy students, Chesteron et al., (2003) [18] found excellent inter-rater reliability (mean ICC: 0.91) using a digital algometer to measure PPTs on the first dorsal interossei. The raters in our study had a variety of years of post-graduate clinical experience but minimal experience using algometry and undertook a single one-hour training session with a brief ‘top-up’ prior to testing. Our results agree with that of Walton et al., (2011) [15] that this time frame is enough to train raters to use pressure algometry to acceptable reliability in a clinical environment. However, the lower inter-rater reliability suggests further training would be appropriate in sporting environments where several members of the sports medicine team are involved in player assessment and rehabilitation (e.g., elite football teams). Further research could therefore investigate whether the amount of training effects the reliability of PPT assessment.

### 4.2. Bland–Altman Plots and Standard Error of Measurement

In a previous study, higher PPT values were associated with greater measurement area [15]. However, visual inspection of the Bland–Altman plots in our suggests that the amount of pressure did not influence the amount of measurement error. Therefore, the algometer can be used at higher levels but this does not agree with previous studies. Linear regression suggests that all Bland–Altman plots were free of proportional bias apart from intra-rater testing of the Sural nervous tissue. The SEM values ranged between 3.47 to 5.62 kPa, and since these were lower than the SD for all points, for all raters, the results are unlikely to have occurred due to measurement error [26]. Therefore, overall, the study supports the use of pressure algometer for assessing the PPTs of the Sural and Tibial nervous tissue at the ankle in elite youth male footballers.

### 4.3. PPT Values at the Ankle Nervous Tissue versus Other Anatomical Locations

The PPT values in our study at the Tibial (34.00–47.47 kPa) and Sural (31.59–50.23 kPa) nervous tissue were lower than those in Fingleton et al., (2014) [11] (124–141 kPA) and Walsh & Hall et al., (2009) [12] (149–174 kPa). These values could be explained by the ankle having less surrounding soft-tissue and the adjacent neural tissue being more superficial than the hip or knee. Furthermore, elite footballers have relatively very low body fat scores compared to non-sporting populations [27].

Conversely, Sterling et al. [28] found comparatively higher PPT values at the nervous tissue around the elbow. Although exact values were not reported, visual inspection of the graphs provided in Sterling et al., (2000) suggests that PPT ranges were between >200 to approximately 400 kPa across the median, radial, and ulna nerves respectively. Even while acknowledging some error from visually inspection of the graphs, these figures are substantially higher than the PPT values found in our study.

The study populations of Fingleton et al., (2014) [11], Walsh & Hall et al., (2009) [12] and Sterling et al., (2000) [28] were all ≥18 years of age. Although no study provided sufficient detail to relate specific age profiles to PPT values, adolescents have been reported to have reduced pain thresholds related to ‘growing pain’ [29]. Therefore, it is possible that our lower PPT values were also related to our study population.

### 4.4. Study Limitations

Although we assessed intra-rater and inter-rater reliability, no data was collected on test–retest reliability. Therefore, no data exists on the algometers usefulness to measure changes in PPTs within a treatment session. The population in our study were limited to asymptomatic male footballers from an elite academy with a narrow age range. As evidence suggests that gender and age have a significant effect on pain perception [17], further testing is required on female football players, players of different levels, and those who have suffered lower-limb musculoskeletal injury to verify if these results are generalisable.

Due to difficulties with location during training, the Superficial Peroneal nervous tissue was excluded and from testing and analysis and, therefore, recommendations on its reliability of PPT assessment cannot be made. As the Superficial Peroneal nervous tissue is likely to become sensitised in players with ankle injuries, this limits the clinical application of our study and warrants further investigation in future studies.

The identification of testing points in our study was made by a non-testing fourth researcher. Although this was done to enhance the accuracy of point location across participants, the reliability of this method was not assessed. Therefore, the results only reflect the inter-rater reliability of handling of the algometer and not point location which may further limit the algometers clinical value.

Finally, although a sample size calculation was done a prior to testing, no normative data of the PPT values of elite male youth footballers exists for comparison and our sample was not large enough to make strong clinical recommendations. Therefore, further PPT testing on larger sample sizes is required.

## 5. Conclusions

The Wagner algometer can be used reliably to assess the PPTs of the Tibial and Sural nervous tissue at the ankle in elite youth male football players. Further testing is required on the reliability of PPT values of the ankle nervous tissue to verify these results. Future studies should include greater sample sizes and focus on the Superficial Peroneal nervous tissue, female footballers, players of different levels, and those who have suffered lower-limb musculoskeletal injury.

## Figures and Tables

**Table 1:** Participant Eligibility Criteria.

Inclusion Criteria	Exclusion Criteria
Elite youth male footballer at the Premier League Football Club in the under 18 s squad Currently participating in full training	Unable to understand written/verbal English Spinal or lower-limb injury in past 12 months Analgesic use with in past 7 days

**Table 2:** Participant Characteristics.

Number of Participants	34
Age (years)	17 ± 1
Age range (years)	16–18
Height (cm)	177.0 ± 3.0
Body mass (kg)	71.0 ± 2.6

Legend: cm, centimetres; kg, kilograms.

**Table 3:** Pain Pressure Values recorded by raters.

Rater/Nerve	Minimum	Maximum	Mean	Standard Deviation
R1/Tibial, T1	24.00	62.00	36.74	7.58
R1/Sural, T1	27.00	72.00	47.47	9.52
R1/Tibial, T2	27.00	70.00	39.12	9.94
R1/Sural, T2	30.00	89.00	50.23	13.84
R2/Tibial	20.00	50.00	38.09	7.61
R2/Sural	20.00	65.00	41.12	11.40
R3/Tibial	18.00	55.00	34.00	8.62
R3/Sural	18.00	48.00	31.59	7.94

Legend: R1, Rater 1; R2, Rater 2; R3, Rater 3; T1: Time point 1—intra-rater testing only; T2: Time point 2—intra-rater tester only; All values displayed as kPa.

**Table 4:** Intra-rater and Inter-rater reliability of Pain Pressure Thresholds.

Intra-Rater Reliability	ICC (95% CI)	SEM	MDC
Tibial	0.88 (0.76, 0.94)	3.47	9.6
Sural	0.89 (0.79, 0.95)	4.24	11.8
Inter-rater reliability			
Tibial	0.66 (0.40, 0.82)	4.69	13.0
Sural	0.71 (0.50, 0.85)	5.62	15.7

Legend: CI, confidence interval; ICC, intraclass correlation coefficient; SD, Standard deviation; SEM, standard error of measurement; MDC, Minimal Detectable Change. SEM and MDC values displayed at kPa.

## Data Availability

The data presented in this study are openly available on FigShare at https://doi.org/10.6084/m9.figshare.15073800.v1.(accessed on 10 September 2021).

## Supplementary Materials

The following are available online at https://www.mdpi.com/article/10.3390/sports9090132/s1, Figure S1: Bland–Altman plots and linear regression for Intra-rater and Inter-rater reliability of Tibial and Sural nervous tissue for elite youth football players.
