# Supplementary material for: Intra-Rater and Inter-Rater Reliability of Pressure Pain Algometry of the Sural and Tibial Nerves in Asymptomatic Elite Youth Footballers
Source: Sports (Basel). 2021 Sep 18;9(9):132. doi: 10.3390/sports9090132 (PMC8472704; doi:10.3390/sports9090132)
Supplement: Supplementary file 1 [file sports-09-00132-s001.zip › sports-1339843-supplementary.pdf]

Figure S1: Bland-Altman plots for Intra-rater and Inter-rater reliability of Tibial and Sural nervous tissue for elite youth football players.

<table>
<tr><td>Tibial</td><td>Sural</td></tr>
<tr><td>Intra-rater Reliability</td><td></td></tr>
<tr><td>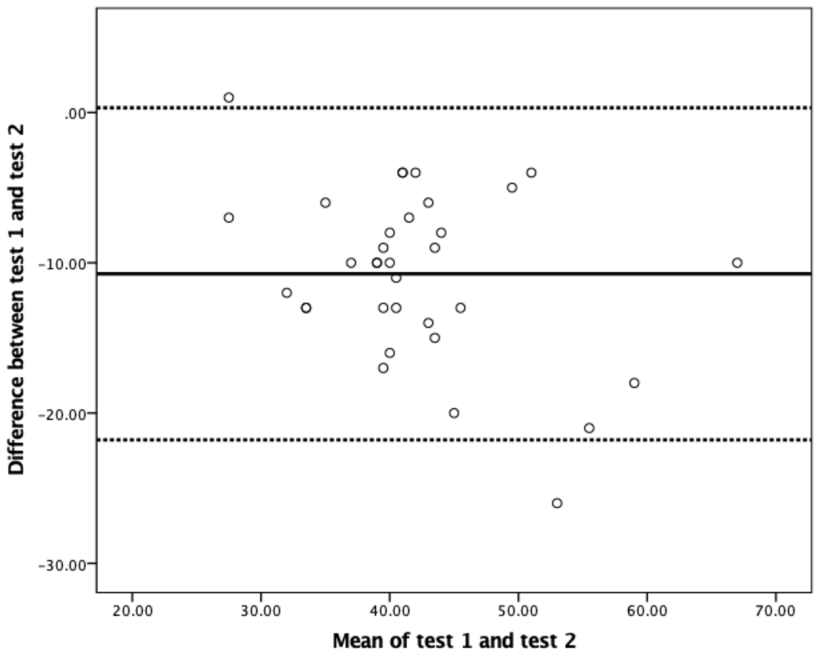

Linear regression value: 0.252</td><td>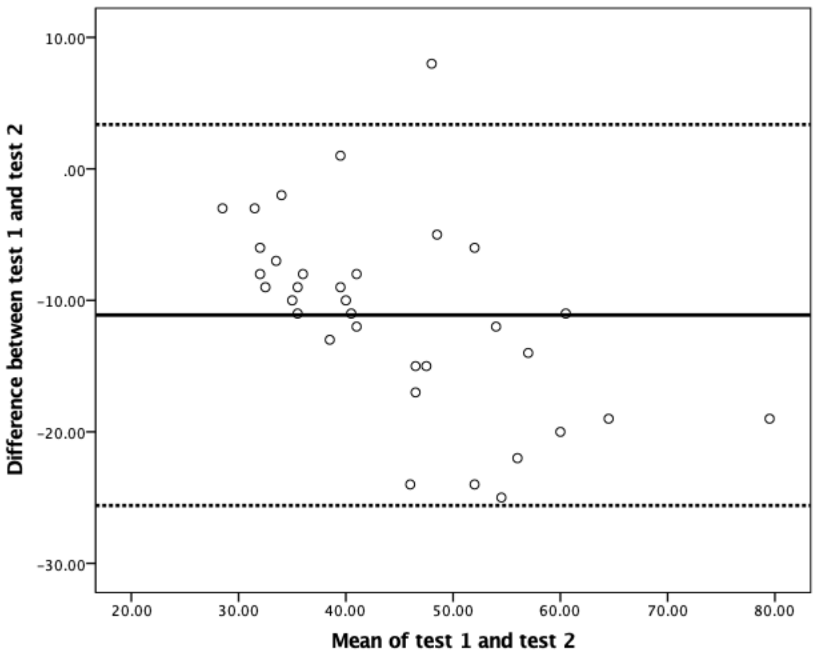

Linear regression value: 0.01</td></tr>
<tr><td>Inter-rater Reliability</td><td></td></tr>
<tr><td>Rater 1 vs. Rater 2</td><td></td></tr>
</table>

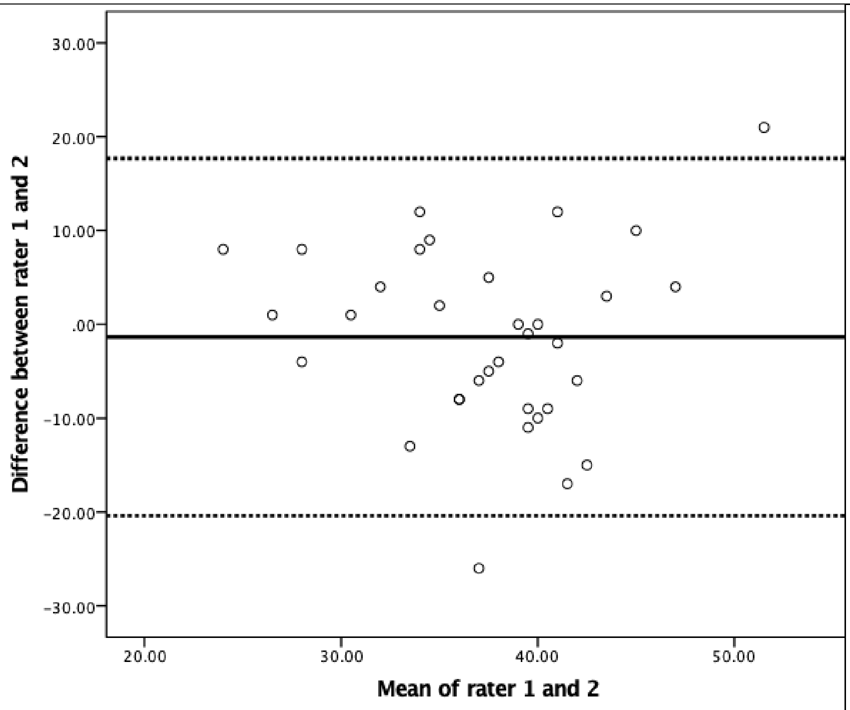 **Linear Regression Value: 0.951**	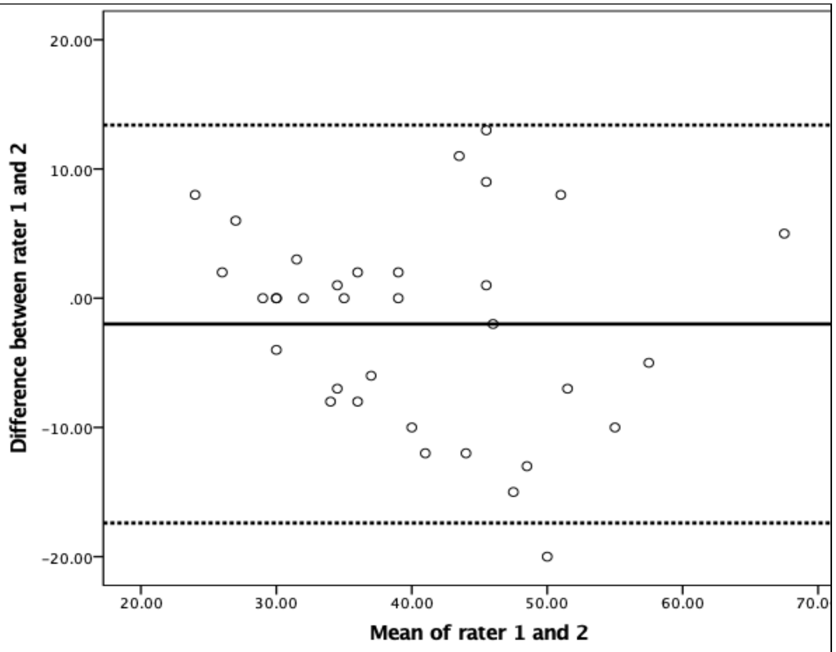 **Linear Regression Value: 0.261**
**Rater 1 vs. Rater 3**	

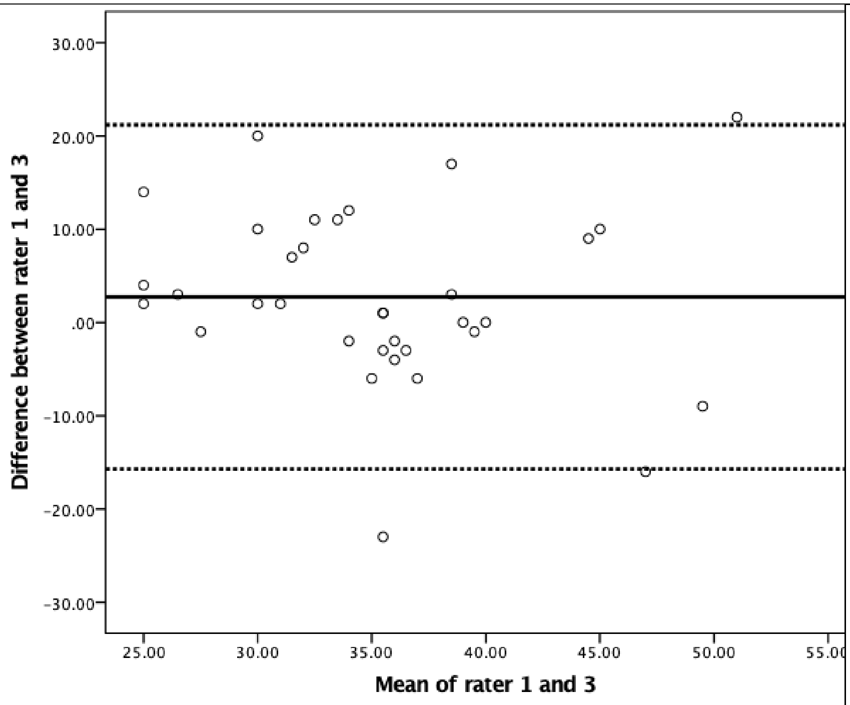

Linear Regression Value: 0.600

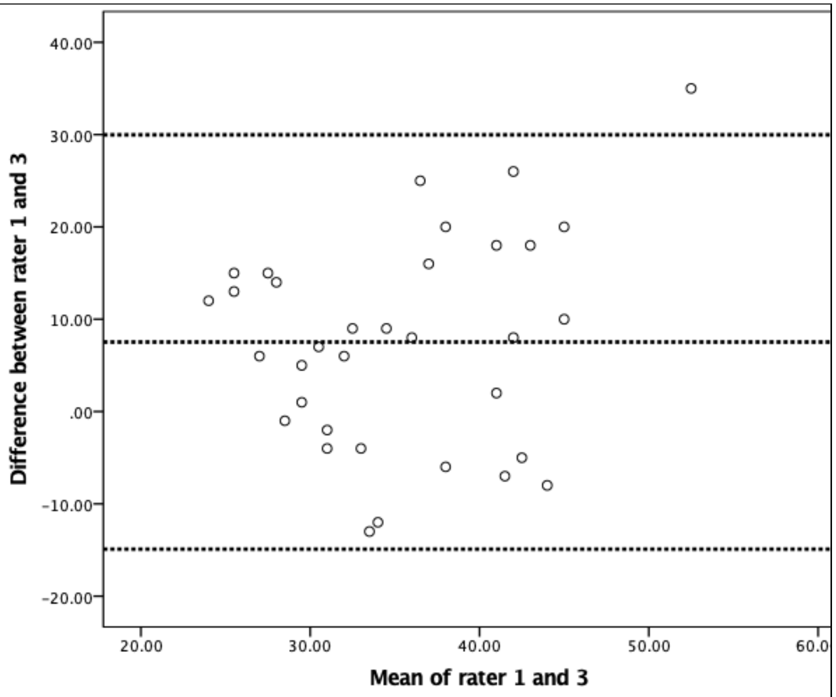

Linear Regression Value: 0.199

Rater 2 vs. Rater 3

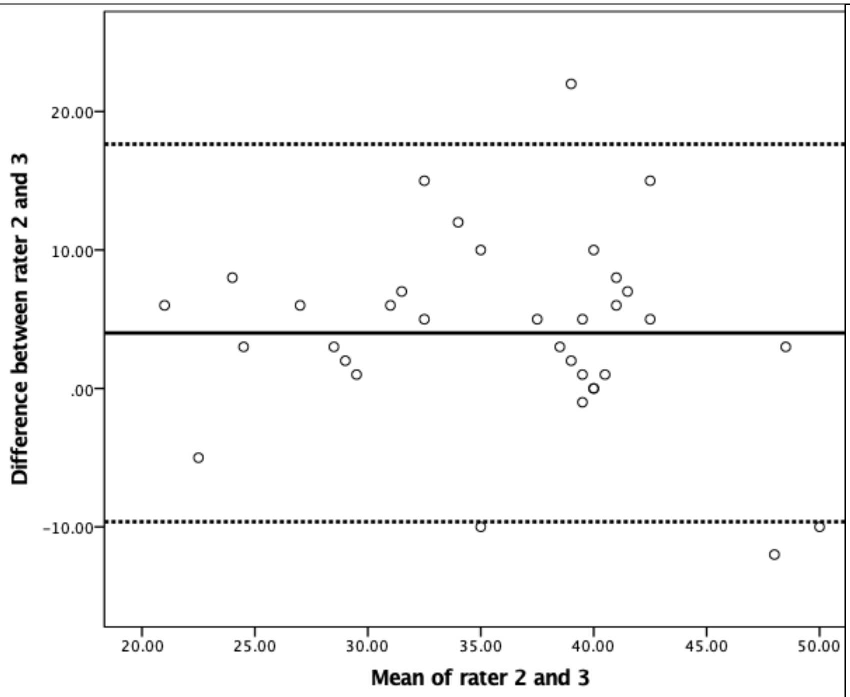

Linear Regression Value: 0.457

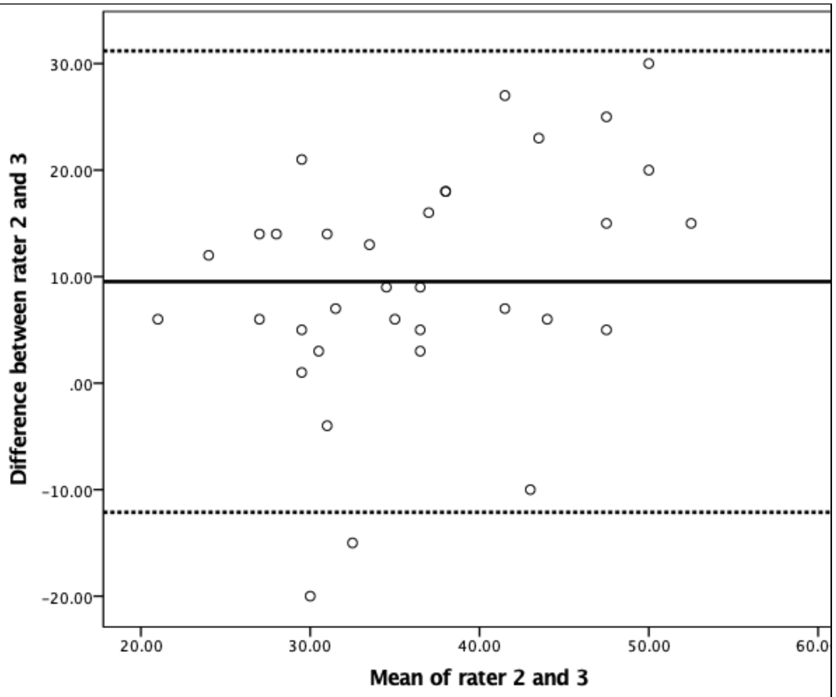

Linear Regression Value: 0.06
